# Trustworthy COVID-19 Mapping: Geo-spatial Data Literacy Aspects of Choropleth Maps

**DOI:** 10.1007/s42489-020-00057-w

**Published:** 2020-10-23

**Authors:** Carsten Juergens

**Affiliations:** Geomatics Group, Institute of Geography, Faculty of Geosciences, Ruhr University Bochum, 44780 Bochum, Germany

**Keywords:** Data literacy, Thematic map, COVID-19, Corona pandemic, Mapping, Big data, Choropleth map

## Abstract

Since the COVID-19 (coronavirus disease 2019) pandemic is a global phenomenon, many scientists and research organizations create thematic maps to visualize and understand the spatial spread of the disease and to inform mankind. Nowadays, Geographic Information Systems (GIS) and web mapping technologies enable people to create digital maps on demand. This fosters the permanent update of COVID-19 map products, even by non-cartographers, and their publication in news, media and scientific publications. With the ease and speed of map-making, many map creators seem to forget about the fundamental principles of good and easy-to-read thematic choropleth maps, which requires geo-spatial data literacy. Geo-spatial data literacy is an important skill, to be able to judge the reliability of spatial data, and to create ingenuous thematic maps. This contribution intends to make people of disciplines other than those that are map-related aware of the power of thematic maps and how one can create trustworthy thematic maps instead of misleading thematic maps which could, in a worst case,  lead to misinterpretation.

## Abstract (de)

Da die Pandemie COVID-19 (Coronavirus-Krankheit 2019) ein globales Phänomen ist, erstellen viele Wissenschaftlerinnen und Wissenschaftler sowie Forschungsorganisationen thematische Karten, um die räumliche Ausbreitung der Krankheit zu visualisieren und zu verstehen und um die Menschheit darüber zu informieren. Heutzutage ermöglichen Geographische Informationssysteme (GIS) und Web-Mapping-Technologien kurzfristig die bedarfsgerechte Erstellung digitaler Karten. Dies fördert die ständige Aktualisierung von COVID-19-Kartenprodukten, auch von Nicht-Kartographen, und ihre Veröffentlichung in Nachrichten, Medien und wissenschaftlichen Publikationen. Angesichts der Leichtigkeit und Schnelligkeit der Kartenerstellung scheinen viele Kartographen die Grundprinzipien guter und leicht lesbarer thematischer Choroplethenkarten zu vergessen, was Geo-Datenkompetenz erfordert. Geo-Datenkompetenz ist eine wichtige Fähigkeit, um die Glaubwürdigkeit von Geodaten beurteilen zu können und vertrauenswürdige thematische Karten erstellen zu können. Mit diesem Beitrag sollen Menschen anderer als kartenbezogener Disziplinen auf die Einflußmöglichkeiten thematischer Karten aufmerksam gemacht werden und darauf, wie man verläßliche thematische Karten erstellen kann, anstelle von irreführenden thematischen Karten, die im schlimmsten Fall zu Fehlinterpretationen führen könnten.

## Introduction

GIS and web-mapping technologies play an essential role to provide rapid visualization of the geo-spatial spread of the SARS-CoV-2/COVID-19 pandemic (e.g. Zhou et al. 2020; Rosenkrantz et al. 2020). The resulting thematic maps from GIS analysis are used for professionals to identify regional hotspots as well as to find geo-spatial strategies against the pandemic. Besides this, those map products are also published in news and media for the public. Since the public is not trained to read thematic maps critically, it is crucial to visualize the pandemic with easy-to-understand choropleth maps. Unfortunately, often the map-making principles seem to be forgotten while dealing with exciting big data sets. However, cartographers have conducted research on the readability and the usability of thematic maps for decades, and they have found many rules for suitable choropleth maps that avoid to misinform or misguide the map reader (e.g., Bertin 1967; Dibiase et al. 1992; MacEachren 1995; Tyner 2010). This paper is intended to explain shortcomings of existing COVID-19 map examples and to assist in trustworthy choropleth map creation.

## Geo-spatial Data

Geo-spatial data  is composed of descriptive/thematic content (attributes characterizing a spatial entity) and spatial components (e.g., coordinates/coordinate system and geometry). Due to their source and nature, one can distinguish two different data models representing objects of the real world: raster data and vector data (Pászto et al. 2020; Juergens 2020). While raster data represent real-world objects by a number of raster cells with a certain cell size, vector data represent the same objects by points, lines or polygons. For many map products, vector data are used because of their easier handling and smaller file sizes.

## Geo-spatial Data Literacy

The cartographer Monmonier became famous for his publication titled “How to Lie with Maps” (Monmonier 1996). This provoking title aims to make map users aware of the power of cartographers while creating maps. There are many options to influence the information content and the appearance of a thematic map and its readability, e.g., by choosing colors, line styles, text styles, symbol styles or its scale (Juergens 2020). A systematic list and description of visual map variables was developed by Bertin (1967) with seven visual variables (position, size, shape, orientation, color, value, texture) that was extended by Morrison (1974) to nine visual variables and even more extended to an even more pronounced list by MacEachren (1995) with twelve visual variables [(1) location, (2) size, (3) shape, (4) orientation, (5) color hue, (6) color value, (7) texture, (8) color saturation, (9) arrangement, (10) crispness, (11) resolution, and (12) transparency] (Roth 2017).

This paper intends to raise the reader´s awareness against the influencing factors that can distort the information content of choropleth map data. Juergens (2020) describes the geo-spatial data literacy aspects in detail and illustrates many influencing factors. Mooney and Juhász (2020) as well as Griffin (2020) illustrate the power of thematic maps and how one can create trustworthy thematic maps instead of misleading thematic maps which could in a worst case be used for misinterpretation. According to Mooney and Juhász (2020) the cartographer Monmonier (1989) was among the first scientists to create guidelines for the use of thematic maps in journalistic contexts. Very often media use choropleth maps that illustrate each spatial area or region shaded in proportion to a statistical attribute or variable.

In the following sub-sections, one finds an exemplary list of major influencing factors of (choropleth) map-making. Some subsections belong to maps in general, others underline the specific nature of choropleth maps.

### Level of Generalization

To display real-world objects with their coordinates in map representations, one needs some abstraction and simplification of the complex real world. Cartographers generalize the complexity of the real world and create models for a reduced map content. There is a great variety of those techniques; one thing all of them have in common: the cartographer is able to influence the resulting content of the map product (Juergens 2020).

### Map Projection

**Fig. 1:**
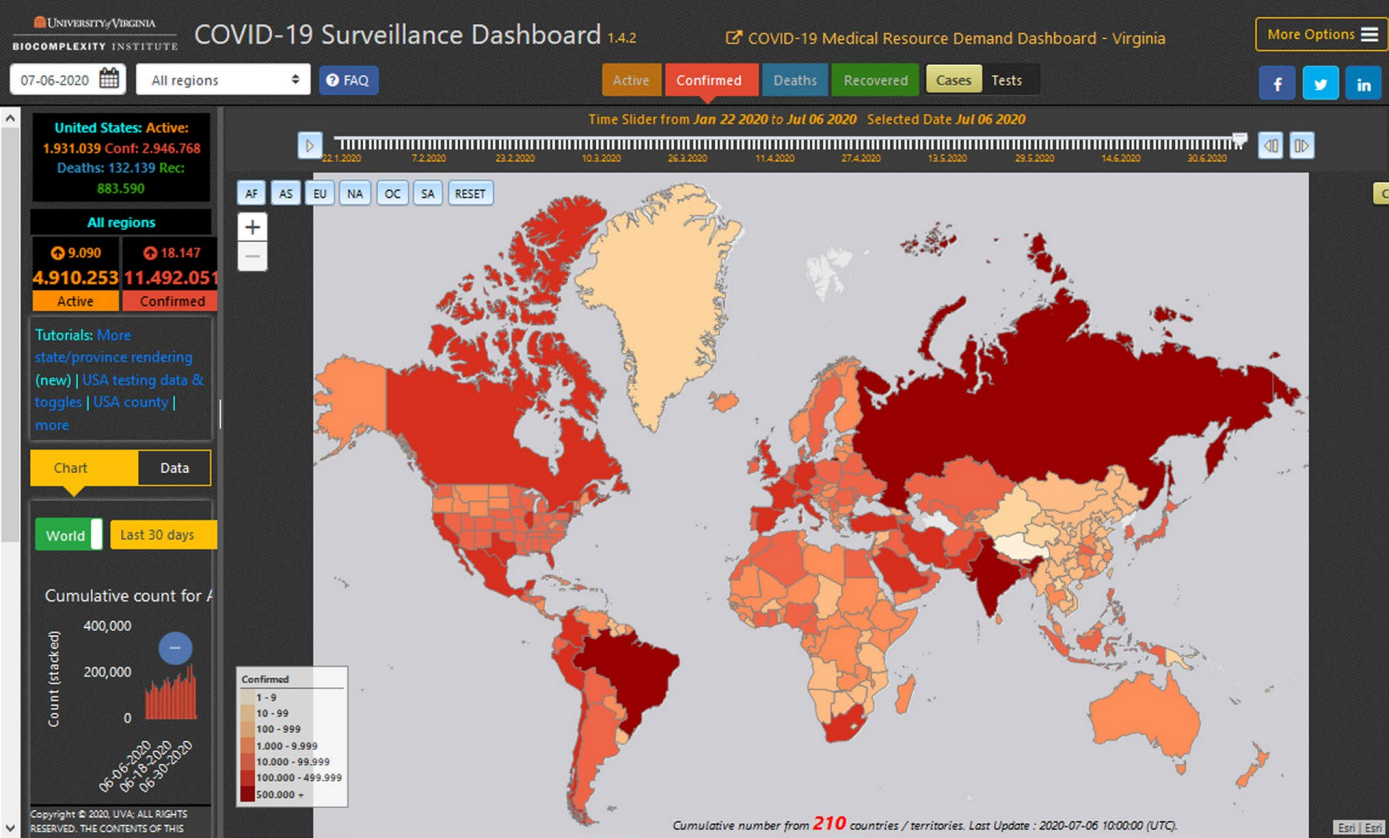
Misleading areal representations of countries due to Mercator Projection (University of Virginia 2020)

A map projection is needed to transform the real world of our 3D globe to a flat 2D map representation. Since map projections are a simplified representation of the true environment, they cause distortions in distance, direction and/or area. For the visualization of global phenomena, very often one finds the Mercator projection (Fig. 1) used for the base map. The disadvantage of this projection is the scale distortion towards the higher latitudes and the resulting misleading appearance of areas (Juergens 2020). For instance, Greenland appears in about the same size as the African continent. In reality, Africa is about 14 times larger. Furthermore, Russia seems to be four times as large as the United States of America (USA). In reality, Russia is only twice the area of the USA.

The problem […] can be overcome with equal area projections, e.g., the Equal Earth projection, which considers the areal complexity and distortion to the north/south and allows the relative comparison of areas. Of course, there are many other projections available to properly visualize the earth’s surface in different scales (Maling 1992). For comparisons of geographical features, one should use maps with the same projection so to not have the distortion errors due to different projections that could result in misleading interpretations (Juergens 2020).

### Scale or Level of Detail

**Fig. 2:**
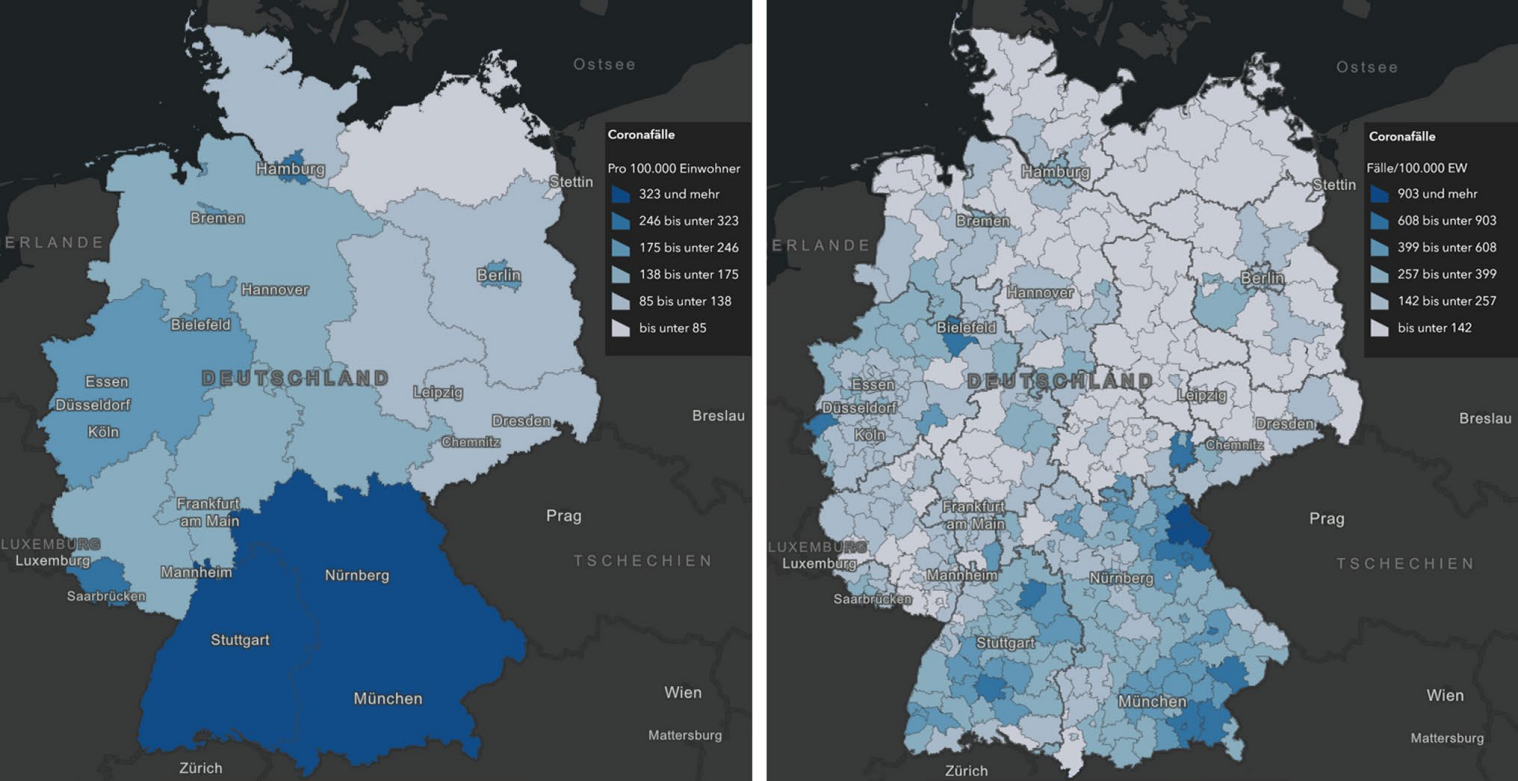
Thematic map of COVID-19 cases/100,000 inhabitants in Germany on July 6, 2020 based on states (left) and counties (right) (Robert Koch Institut 2020) (Legend shows color and value ranges per class). The map based on states is more generalized than the county map that represents a better regional differentiation

Scale is always connected with spatial resolution or level of detail in a study using geo-spatial data. For small-scale maps, a map generalization process is required to reduce the semantic details, since too much semantic detail makes the map unreadable. In addition to that, it makes a difference if a data set is used on a coarse (e.g., small scale) administrative level (such as countries or states within a country) or a finer (e.g., large scale) spatial differentiation (such as city or county border lines). The spatial aggregation influences the information content of map products. In Fig. 2, one can compare the COVID-19 cases/100,000 inhabitants in Germany on July 6, 2020 by administrative levels of different hierarchy: on the administrative level of states and on the finer administrative level of cities and counties. Please note the different legends (same colors but different value ranges) due to different regional scale of the geo-spatial reference layer. In the state, the number of COVID-19 cases/100,000 inhabitants is much lower due to smoothing effects compared to the county layer, where one can much better identify regional hot spots.

### Classification Method of Choropleth Maps

**Fig. 3:**
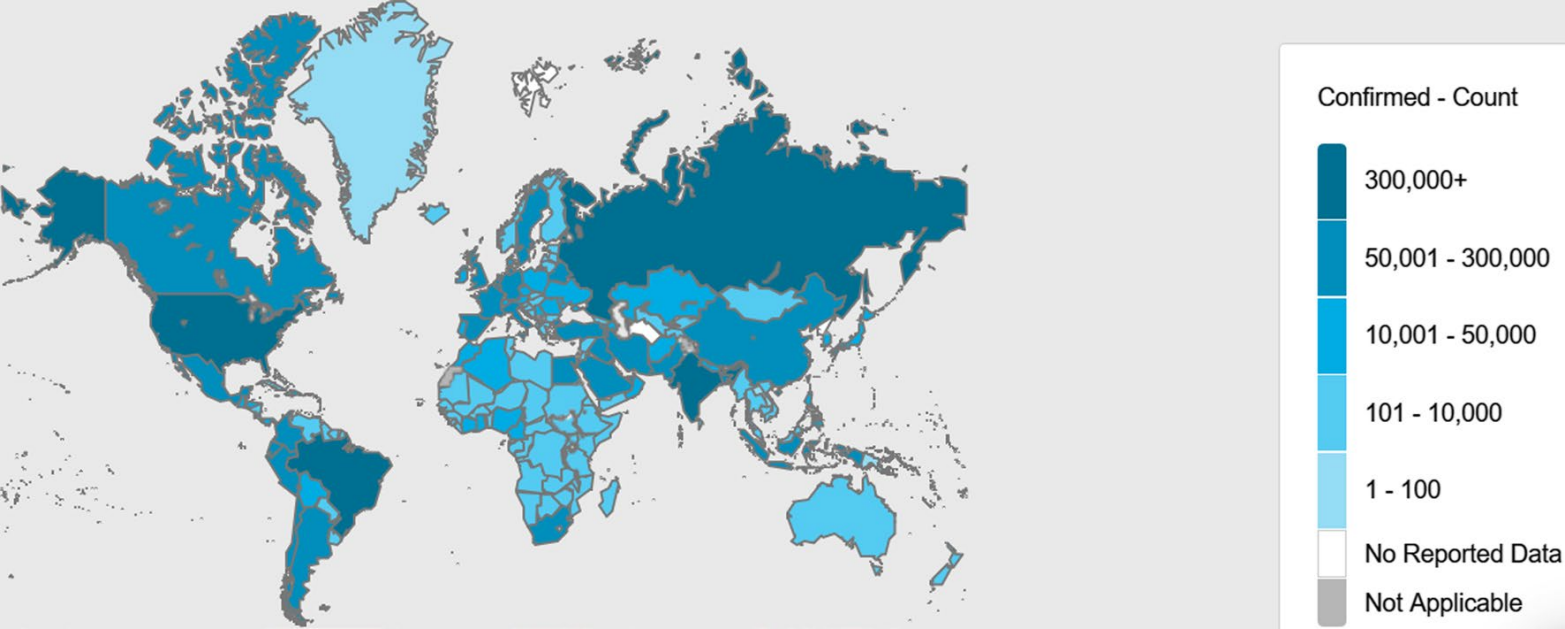
Confirmed Corona cases on July 6, 2020 (World Health Organization 2020)

For thematic maps, a cartographer or geo-information expert has many options to influence the appearance of the resulting map (Kraak and Ormeling 2020). Among those options are the number of thematic classes, the class limits, the classification method to define those classes and the color scheme applied (and its associations at the map readers side) (compare Brewer 2005). Referring to the number of classes, too many classes could lead to a resulting map that looks complex and eventually confusing to the map reader. On the other hand, too few classes could result in a map that is oversimplified and could eventually hide information (Campbell and Shin 2011). As a rule of thumb for an effective classification, one can define approximately 4–6 classes. To illustrate such effects, please compare Figs. 1 and 3. They have different number of classes 7 vs. 5), different color scheme (red/orange vs. blue) and different class limits resulting in a different appearance that would lead to different interpretation results. In addition to that, Fig. 1 does not represent the USA as a country, instead the individual states are shown. The same is done with mainland China, with provinces instead of one color for the country. This results in irritating map classes, since absolute numbers are given. Compared to countries, provinces and states have a relatively smaller absolute number of cases, so one gets the impression that USA and China are not so much affected by the pandemic. Figure 3 shows a clearer picture of the situation of the same day (July 6, 2020) with cumulated number of cases for complete countries only.

### Map Design

Map design is the art of adequately communicating the results of a spatial analysis to the map reader. This is connected to the readability of maps (e.g., colors, symbology, map layout, fonts, etc.) and could also affect the intuitive interpretation of a map (e.g., cultural color semantics). By doing so, one has to be careful not to distort information by the selected map design while creating maps. “[…] a strong working knowledge of cartographic rules will not only assist in the avoidance of potential misrepresentation of spatial information, but also enhance one’s ability to identify these indiscretions in other cartographers’ creations”. (Campbell and Shin, 2011: 210). In Monmonier (1996), cartographic principles are discussed and how maps can be used and misused is illustrated. In Monmonier (1996) and Campbell and Shin (2011), one finds elaborate descriptions of the intricacies of color use, symbol selection and map design as well as layout issues.

**Fig. 4:**
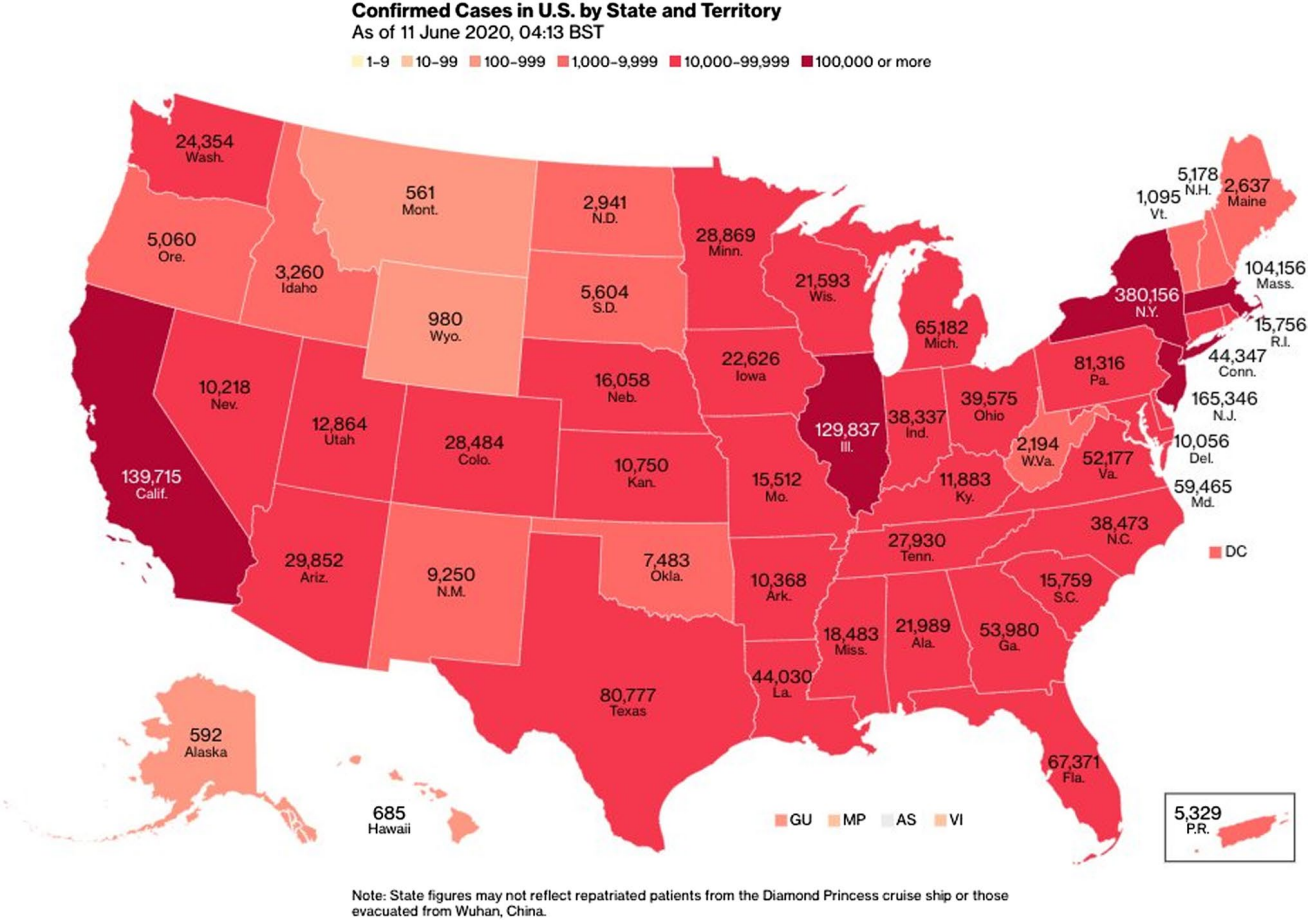
Example of a misleading map with absolute numbers of confirmed COVID-19 cases and predominant red colors (Twitter (2020)

In Fig. 4 also absolute numbers of confirmed COVID-19 cases were used for differently sized US states with varying population. To make the COVID-19 cases comparable, one has to normalize them against the total population. A widely used rate is cases per 100,000 inhabitants. Then, comparisons are meaningful, even in less populated states or regions.

In addition to the irritating absolute numbers, Fig. 4 is illustrated with red colors, which is known to be able to cause anxiety, fear or danger on the map reader’s side. So, this is an inappropriate choropleth map representation for the map content related to a pandemic, which is an unpleasant topic by itself. Slocum et al. (2009) present examples of appropriate choropleth maps and could be consulted for best practice examples.

## Conclusion

Choropleth maps are used for effective visual communication of spatial phenomena. “An essential purpose of choropleth maps is the visual perception of spatial patterns […]. This requires an effective and as intuitive as possible comparison of color values between different regions” (Schiewe 2019). This contribution was supposed to open one’s eyes for the specific nature of choropleth maps. In this context, it was pointed out which factors influence the map appearance, its readability and credibility.

Furthermore, the processing of thematic data is a process with a lot of influencing challenges. The number of classes, the class definition, the classification method and the color scheme can affect the displayed map content strongly.

The major problem for the ‘end-user’ is that one gets final products like maps and one cannot obviously ‘see’ how the product was prepared from source data. A solution could be that end users ask for meta-data that describe data sets and maps. If those are prepared properly, processing steps are described and could be identified by the user. This helps to judge the quality of the product.

Please be aware that maps could be manipulated for specific purposes. One is still able to lie with maps—please consider different interests. The overall goal of this contribution was to make people of other disciplines than geo-related, aware of the characteristics and possible ways of manipulation of maps as well as of their power if applied properly. It is intended to help readers to become more spatially literate people. For more in-depth cartographic research on the specific user perception of choropleth map variables one should perform and evaluate specific user tests to identify the optimal choropleth map composition for a specific applications. For instance, Schiewe (2019) empirically investigated the visual perception of spatial patterns in choropleth maps. Brewer and Pickle (2002) present an early example on the evaluation of methods for classifying epidemiological data on choropleth maps. And finally, Hruby (2016) provides an overview of choropleth maps from the beginning.

## Data Availability

Data sharing is not applicable to this article as no datasets were generated or analyzed during the current study.

## References

[CR1] Bertin J (1967). Sémiologie graphique: Les diagrammes, les réseaux, les cartes.

[CR2] Brewer CA (2005). Designing better maps: a guide for GIS users.

[CR3] Brewer CA, Pickle LW (2002). Evaluation of methods for classifying epidemiological data on choropleth maps in series. Ann Assoc Am Geogr.

[CR4] Campbell JE, Shin M (2011) Essentials of geographic information systems; Saylor Academy: Washington, DC, USA, https://digitalcommons.liberty.edu/textbooks/2. Accessed 6 Jul 2020

[CR5] Dibiase D, MacEachren AM, Krygier JB, Reeves C (1992). Animation and the role of map design in scientific visualization. Cartogr Geogr Inf Syst.

[CR6] Griffin AL (2020). Trustworthy maps. J Spatial Inf Sci.

[CR7] Hruby F (2016). 190 Jahre Choroplethenkarte—Ein Zwischenresümme. KN J Cartogr Geogr Inf Heft.

[CR8] Robert Koch Institut. COVID-19-Dashboard (2020) https://experience.arcgis.com/experience/478220a4c454480e823b17327b2bf1d4. Accessed 6 Jul 2020

[CR9] Juergens C (2020). Digital data literacy in an economic world: geo-spatial data literacy aspects. ISPRS Int J Geo-Inf.

[CR10] Kraak MJ, Ormeling FJ (2020). Cartography.

[CR11] MacEachren AM (1995). How maps work.

[CR12] Maling DH (1992). Coordinate systems and map projections.

[CR13] Monmonier M (1989). Maps with the news: the development of American journalistic cartography.

[CR14] Monmonier M (1996). How to lie with maps. Am Stat.

[CR15] Mooney P, Juhász L (2020). Mapping COVID-19: how web-based maps contribute to the infodemic. Dialogues Hum Geogr.

[CR16] Morrison JL (1974). A theoretical framework for cartographic generalization with the emphasis on the process of symbolization. Int Year Book Cartogr.

[CR17] Pászto V, Redecker A, Macků K, Jürgens C, Moos N, Pászto V, Jürgens C, Tominc P, Burian J (2020). Data sources. SPATIONOMY—spatial exploration of economic data and methods of interdiscyplinary analytics.

[CR18] Rosenkrantz L, Schuurman N, Bell N, Amram O (2020). The need for GIScience in mapping COVID-19. Health Place.

[CR19] Roth RE, Richardson D, Castree N, Goodchild MF, Kobayashi A, Liu W, Marston RA (2017). Visual variables. International encyclopedia of geography: people, the earth, environment and technology.

[CR20] Schiewe J (2019). Empirical studies on the visual perception of spatial patterns in choropleth maps. KN J Cartogr Geogr Inf.

[CR21] Slocum TA, McMaster RB, Kessler FC, Howard HH (2009). Thematic cartography and geovisualization.

[CR22] Twitter (2020) Mapping where coronavirus is spreading across the U.S. 2020. https://twitter.com/business/status/1271073464003198976. Accessed 6 Jul 2020

[CR23] Tyner JA (2010). Principles of map design.

[CR24] University of Virginia (2020) COVID-19 surveillance dashboard. https://nssac.bii.virginia.edu/covid-19/dashboard/. Accessed 6 Jul 2020

[CR25] World Health Organization (2020) WHO coronavirus disease (COVID-19) dashboard. https://covid19.who.int/. Accessed 6 July 2020

[CR26] Zhou C, Su F, Pei T, Zhang A, Du Y, Luo B, Cao Z, Wang J, Yuan W, Zhu Y, Song C, Chen J, Xu J, Li F, Ma T, Jiang L, Yan F, Yi J, Hu Y, Liao Y, Xiao H (2020). COVID-19: challenges to GIS with big data. Geogr Sustain.

